# Predicting mental health–related sick leave using stress check data: development and validation of a workplace risk model

**DOI:** 10.1093/joccuh/uiag011

**Published:** 2026-02-26

**Authors:** Shunsuke Inoue, Tomohisa Nagata, Naoto Ito, Yuka Takahashi, Yusuke Hoshi, Mika Sato, Masahide Minami, Masayoshi Ida, Koji Mori

**Affiliations:** Department of Occupational Health Practice and Management, Institute of Industrial Ecological Sciences, University of Occupational and Environmental Health, Japan, 1-1 Iseigaoka, Yahatanishi-ku, Kitakyushu 807-8555, Japan; Health Promotion Center, Komatsu Ltd., Shiodome Building, 1-2-20, Kaigan, Minato-ku, Tokyo 105-8316, Japan; Department of Occupational Health Practice and Management, Institute of Industrial Ecological Sciences, University of Occupational and Environmental Health, Japan, 1-1 Iseigaoka, Yahatanishi-ku, Kitakyushu 807-8555, Japan; Department of Occupational Health Practice and Management, Institute of Industrial Ecological Sciences, University of Occupational and Environmental Health, Japan, 1-1 Iseigaoka, Yahatanishi-ku, Kitakyushu 807-8555, Japan; Health Promotion Center, Komatsu Ltd., Shiodome Building, 1-2-20, Kaigan, Minato-ku, Tokyo 105-8316, Japan; Health Promotion Center, Komatsu Ltd., Shiodome Building, 1-2-20, Kaigan, Minato-ku, Tokyo 105-8316, Japan; Health Promotion Center, Komatsu Ltd., Shiodome Building, 1-2-20, Kaigan, Minato-ku, Tokyo 105-8316, Japan; Health Promotion Center, Komatsu Ltd., Shiodome Building, 1-2-20, Kaigan, Minato-ku, Tokyo 105-8316, Japan; Health Promotion Center, Komatsu Ltd., Shiodome Building, 1-2-20, Kaigan, Minato-ku, Tokyo 105-8316, Japan; Health Promotion Center, Komatsu Ltd., Shiodome Building, 1-2-20, Kaigan, Minato-ku, Tokyo 105-8316, Japan; Department of Occupational Health Practice and Management, Institute of Industrial Ecological Sciences, University of Occupational and Environmental Health, Japan, 1-1 Iseigaoka, Yahatanishi-ku, Kitakyushu 807-8555, Japan

**Keywords:** Stress Check program, Brief Job Stress Questionnaire (BJSQ), mental health–related sick leave, prediction model, occupational health, risk stratification

## Abstract

**Objectives:**

To develop and validate a predictive model for mental health–related sick leave using data from Japan’s Stress Check program linked with personnel records (demographics and sick-leave history), and to evaluate its predictive performance compared with the conventional high-stress classification.

**Methods:**

We conducted a retrospective cohort study of employees (2020-2024) in a 14-company corporate group spanning diverse occupations. A mixed-effects logistic regression model was developed using data from 2020-2023, with company and year as random intercepts. Variable robustness was confirmed through least absolute shrinkage and selection operator (LASSO) logistic regression. Model calibration and discrimination were evaluated using the Brier score and area under the curve (AUC), respectively. The model was temporally validated on 2024 data.

**Results:**

Among 87 138 person-years in the development phase, 695 employees took mental health–related sick leave (0.8%). Seven predictors—younger age, anxiety, depression, sleep disturbance, low job control, poor job suitability, and low coworker support—were significantly associated with sick leave. The recalibrated model showed good discrimination (AUC = 0.708) and calibration (Brier score = 0.0079) in development, and higher performance in validation (AUC = 0.819; Brier score = 0.0026). The conventional high-stress classification performed poorly.

**Conclusions:**

The proposed model demonstrated robust predictive validity and outperformed the conventional high-stress classification. By providing quantitative and continuous risk stratification within the Stress Check framework, it offers a practical approach to support risk-based prioritization and decision-making in occupational health practice.

## Teaser

**Key points**

**What is already known on this topic**

Employees classified as “high stress” under Japan’s Stress Check Program have an elevated risk of subsequent long-term mental health–related sick leave. However, the conventional high-stress criterion is a rule-based, binary classification that does not capture the heterogeneity of future risk or allow flexible risk ranking.

**What this study adds**

This study developed and validated a multivariable prediction model using routinely collected Stress Check and personnel data that more precisely stratifies future sick-leave risk and outperforms the conventional high-stress classification.

**How this study might affect research, practice, or policy**

The model enables practical, quantitative risk stratification within the existing Stress Check framework, supporting earlier, more targeted occupational health interventions and more efficient allocation of limited workplace health resources.

## 1. Introduction

Mental health problems among working-age populations impose substantial burdens on individuals, employers, and society through reduced productivity and increased medical and compensation costs.^1,2^ Long-term sick leave due to mental disorders is particularly consequential, as it complicates return to work and increases the risk of relapse, underscoring the need for effective primary prevention.^3–5^ In response, international bodies and national governments have implemented workplace programs to prevent stress-related disorders.^6–10^ In Japan, the Occupational Safety and Health Act was revised in 2015 to mandate an annual Stress Check program for workplaces with 50 or more employees.^11^ Under this system, employers typically use the Brief Job Stress Questionnaire (BJSQ) to identify employees with high stress levels and must offer a physician interview upon the employee’s request.^12^ An expansion of the program to smaller workplaces (fewer than 50 employees) has been proposed, further highlighting the need to leverage Stress Check data more effectively for prevention.^13^

Employees classified as high stress have a substantially higher risk of long-term sick leave than those not classified as high stress.^14^ However, the conventional high-stress classification is determined by an administratively defined, multidimensional rule combining stress responses, job stressors, and social support, rather than by a single continuous scale or clinical diagnosis, resulting in a binary classification of workers. Moreover, because the conventional high-stress criterion is defined by a rule-based combination of multiple domains rather than by a single continuous risk score, it does not allow flexible recalibration or continuous risk ranking, which limits its usefulness for individualized risk stratification. This approach may not sufficiently capture heterogeneity in subsequent sick-leave risk within the high-stress group, limiting its usefulness for guiding targeted preventive occupational health interventions. One potential approach is to evaluate whether routinely collected Stress Check data can stratify future sick-leave risk more precisely than the conventional classification, without requiring additional data collection.

Kawamura et al^15^ developed a BJSQ-based prediction model and compared its performance with the national high-stress criterion among university staff. Their findings suggested the potential value of Stress Check–based risk prediction; however, the generalizability beyond a single occupational sector remains uncertain. We therefore aimed to examine whether routinely collected Stress Check data can be used, through a reproducible modeling process, to stratify future sickness-absence risk beyond the conventional high-stress classification in a real-world setting, within a 14-company corporate group encompassing diverse occupations.

## 2. Methods

### 2.1. Study design and sample

We conducted a retrospective cohort study using routinely collected Stress Check and personnel records (demographics and sick-leave history) and reported it in accordance with the TRIPOD statement for prediction model development and validation (Supplementary Digital Content).^16^ Employees from Company A Group’s domestic sites who completed the annual Stress Check between January 1, 2020 and December 31, 2024 were eligible. Company A Group consists of 14 affiliated companies centered on a comprehensive machinery manufacturer (construction/mining and industrial machinery), with additional entities such as a semiconductor equipment maker and training schools, and includes diverse occupations (research and development [R&D], production line, production support, sales/service, administrative, and others). Stress Checks were conducted annually by employers for eligible employees, in accordance with the Occupational Safety and Health Act. The dataset comprised Stress Check and personnel records collected annually from 2020 through 2024. These data were later used for model development and validation. To minimize potential sources of bias, all data were collected through routine annual surveys rather than self-selection, and clustering by company and year was accounted for using random intercepts. Missing data were handled using complete-case analysis; records with missing values in key predictors or outcome variables were excluded from model development and validation.

The study was approved by the Ethics Committee of the University of Occupational and Environmental Health, Japan (approval No. ER25-037). We used anonymized data collected through company health programs with information provided to employees and an opt-out option.

### 2.2. Outcome

The outcome was any episode of mental health–related sickness absence lasting at least 1 month during the study period (“sick leave”). The 1-month threshold aligns with the national Occupational Safety and Health Survey. Eligible cases were determined by treating or occupational physicians as long-term absence primarily due to mental health reasons. For employees with multiple episodes, only the first episode was analyzed. For each annual Stress Check, employees were followed for up to 1 year from the date of assessment, and only sickness absence occurring within this follow-up window was considered as the outcome. Although data from multiple years were used for model development, the prediction target was consistently defined as sickness absence within 1 year following each annual Stress Check.

### 2.3. Predictors and covariates

Predictors comprised 17 BJSQ subscales: 9 from the 23-item version—quantitative job overload, job control, fatigue, anxiety, depression, loss of appetite, sleep disturbance, supervisor support, and coworker support—and 8 from the 57-item version—qualitative job overload, physical demands, skill utilization, interpersonal conflict, poor physical environment, job suitability, meaningfulness of work, and job satisfaction. Under Japan’s Stress Check program, both the 57-item and 23-item versions of the BJSQ are officially recommended. The 23-item version is positioned as a short-form screening instrument intended to reduce respondent burden while retaining key psychosocial constructs relevant to mental health risk. Following the Ministry of Health, Labour and Welfare (MHLW) manual, all scales were coded so that higher scores indicate worse status. Conventional high-stress classification was based on the official criteria of the MHLW using the 23-item short-form BJSQ and the summed-score method. High stress was defined if either of the following criteria was met: (1) the summed score of stress responses (11 items) was ≥31; or (2) the summed score of job stressors and social support (12 items) was ≥39 and the summed score of stress responses was ≥23. All items were rated on a 4-point scale, with higher scores indicating higher stress. The specific questionnaire items and scale composition are provided in Table S1. Covariates were age, sex, and occupation category (R&D, production line, production support, sales/service, administrative, other). For non-cases, all available Stress Check results from 2020 to 2024 were retained. For cases, Stress Check responses obtained before the start of sick leave were used; post-onset responses were excluded. For example, if sick leave began after the 2022 Stress Check, we used 2020-2022 data and excluded 2023 onward. For employees who went on sick leave in 2020 but did not complete the 2020 Stress Check, we exceptionally used their 2019 result to capture pre-onset status.

### 2.4. Statistical analysis

We developed and validated a sick-leave risk-prediction model through a development phase (2020-2023) and a temporal validation (2024) phase.

In the development phase, we fitted a mixed-effects logistic regression as the primary model, with sick leave (yes/no) as the dependent variable. Fixed effects included age, sex, occupation, and BJSQ subscales. Random intercepts were specified for company and year (2020-2023) to account for clustering across companies and calendar years. As a sensitivity analysis, we additionally fitted a model including an individual-level random intercept to account for within-person correlation. Multicollinearity was assessed using variance inflation factors (VIFs). Discrimination was evaluated using the area under the receiver operating characteristic curve (AUC). To assess the stability of variable selection, we performed a least absolute shrinkage and selection operator (LASSO) logistic regression using the same set of predictors. The regularization parameter (λ) was selected using k-fold cross-validation. The cross-validated fit was evaluated across a grid of λ values, and the optimal λ was chosen based on cross-validated model performance. Candidate predictors were initially examined in a full mixed-effects logistic regression model. The final prediction model was constructed by selecting variables based on a combination of statistical significance, model stability across modeling approaches (mixed-effects and LASSO), and practical interpretability for occupational health practice. Based on variables retained in either model, we constructed the logit function and computed individual predicted probabilities:


$$logi{t}_{(p)}={\beta}_0+{\beta}_1{X}_1+{\beta}_2{X}_2+\cdots +{\beta}_k{X}_k$$

$$\kern-3.9pc p=\frac{1}{1+{e}^{- logit(p)}}$$

Calibration was assessed by comparing observed and predicted sick-leave rates across deciles of predicted risk. When miscalibration was observed, logistic recalibration was performed using the original *logit_(p)_* as the sole predictor to estimate intercept (*α*) and slope (*β*):


$$logi{t}_{(recal)}=\alpha +\beta\ logi{t}_{(p)}$$

$$\kern1pc p(recal)=\frac{1}{1+{e}^{- logit(recal)}}$$

After recalibration, model performance was evaluated using AUC and the Brier score. Operational thresholds were examined using sensitivity, specificity, precision, recall, the F1 score, and the Youden index (sensitivity + specificity − 1).

For temporal validation, the final model was applied to the 2024 dataset, and predictive performance was assessed using the AUC and Brier score, in comparison with the conventional high-stress classification. We also evaluated cutoff-dependent performance using sensitivity (recall), specificity, precision (positive predictive value), the Youden index (sensitivity + specificity − 1), and the F1 score at prespecified cutoffs. The Youden-optimized and F1-optimized cutoffs were determined in the development dataset and then applied unchanged to the 2024 validation dataset for head-to-head comparison with the conventional high-stress classification.

All analyses were conducted using Stata Statistical Software, Release 19 (StataCorp LLC, College Station, TX, USA). Two-sided *P* values <.05 were considered statistically significant.

## 3. Results

### 3.1. Sample characteristics

During 2020-2024, a total of 109 296 person-years of Stress Check data were available. In the development phase (2020-2023), 87 138 person-years were available and 695 employees experienced the outcome (sick-leave rate, 0.8%), meeting the common rule of more than 10 events per predictor for model development.^17^ In the temporal validation phase (2024), 22 158 employees were included and 57 experienced the outcome (0.26%). Participant characteristics are summarized in Table 1. In both the development and validation phases, employees who took sick leave tended to be younger than non-cases, and production-line workers—the largest occupation group overall—were relatively overrepresented among cases.

**Table 1:** Characteristics of participants by sick-leave status (development and validation datasets).

	Development data		Validation data	
	Sickness absence (*n* = 695)	No sickness absence (*n* = 86 443)	Sickness absence (*n* = 57)	No sickness absence (*n* = 22 101)
**Age, median (IQR)**	38 (29, 48)	42 (32, 50)	35 (24, 46)	42 (33, 52)
**Sex, male, *n* (%)**	579 (83.3)	75 490 (87.3)	45 (79.0)	19 171 (86.7)
**Occupation classification, *n* (%)**				
**Research and development**	144 (19.6)	18 736 (21.4)	10 (17.5)	4712 (21.3)
**Line workers**	166 (22.6)	24 203 (27.2)	16 (28.1)	6104 (27.6)
**Production support staff**	137 (18.6)	14 317 (16.1)	15 (26.3)	3380 (15.3)
**Sales and service**	155 (21.1)	19 301 (21.7)	9 (15.8)	4675 (21.2)
**Clerical and administrative support**	129 (17.6)	10 412 (11.7)	3 (5.3)	2300 (10.4)
**Others**	4 (0.5)	2094 (2.4)	4 (7.0)	930 (4.2)
**High stress, Yes, *n* (%)**	264 (35.9)	10 157 (11.4)	29 (50.9)	2372 (10.7)

Abbreviations: BJSQ, Brief Job Stress Questionnaire; IQR, interquartile range.

High stress was defined according to the official criteria of the Japanese Ministry of Health, Labour and Welfare Stress Check program using the 23-item short-form BJSQ (summed-score method).

### 3.2. Mixed-effects model and predictor selection

To confirm the utility of the hierarchical structure, we compared the mixed-effects logistic model with a single-level logistic model. The mixed-effects specification provided a significantly better fit (likelihood-ratio test: χ^2^(1) = 933.4; *P* < .001). Of the candidate predictors examined in the full mixed-effects model, 7 variables were retained in the final prediction model based on their statistical significance, stability across modeling approaches, and practical interpretability. Full parameter estimates from the initial model are provided in Table S2. In the primary mixed-effects model, younger age, anxiety, depression, sleep disturbance, lower job control, poorer job suitability, and lower coworker support were significantly associated with higher risk of sick leave. Regression results for the final model are presented in Table 2. Assessment of multicollinearity yielded a maximum VIF of 3.24 and a mean VIF of 1.83 across predictors, indicating no concerning collinearity (all VIF < 10). The variables identified in the primary analysis were consistently selected in the LASSO logistic regression, supporting the robustness of variable selection. The estimates from this sensitivity analysis were largely consistent with those from the primary analysis. Detailed results are provided in Table S3.

**Table 2:** Mixed-effects logistic regression predicting mental health–related sick leave.

Predictor	Coefficient	95% CI	*P* value
**Age**	−0.022	−0.031 to −0.014	<.001
**Anxiety**	0.135	0.076 to 0.194	<.001
**Depression**	0.079	0.018 to 0.140	.011
**Sleep disturbance**	0.146	0.041 to 0.250	.006
**Job control**	0.051	0.002 to 0.100	.041
**Job suitability**	0.215	0.068 to 0.363	.004
**Coworker support**	0.059	0.008 to 0.109	.023
**Intercept**	−5.672	−6.707 to −4.637	<.001

Intercept is the log-odds when all predictors are 0 (at the reference categories).

### 3.3. Risk equation

For practical use and visualization, we derived the following logit function based on the 7 predictors that were statistically significant in the primary model:


$${\displaystyle \begin{array}{l} logit(p)=-5.377-0.022\times Age+0.135\times Anxiety+0.079\\\times Depression+0.146\times Sleep {} disturbance+0.051\times Job\ control\\+0.215\times Job\ suitability+0.059\times Coworker\ support.\end{array}}$$

Predicted probabilities were computed as


$$\kern-3.9pc p=\frac{1}{1+{e}^{- logit(p)}}$$

$$logi{t}_{(recal)}=-1.544922+1.005281\times logi{t}_{(p)}$$

$$\kern-4pc {p}_{recal}=\frac{1}{1+{e}^{- logit(recal)}}$$

**Figure 1:**
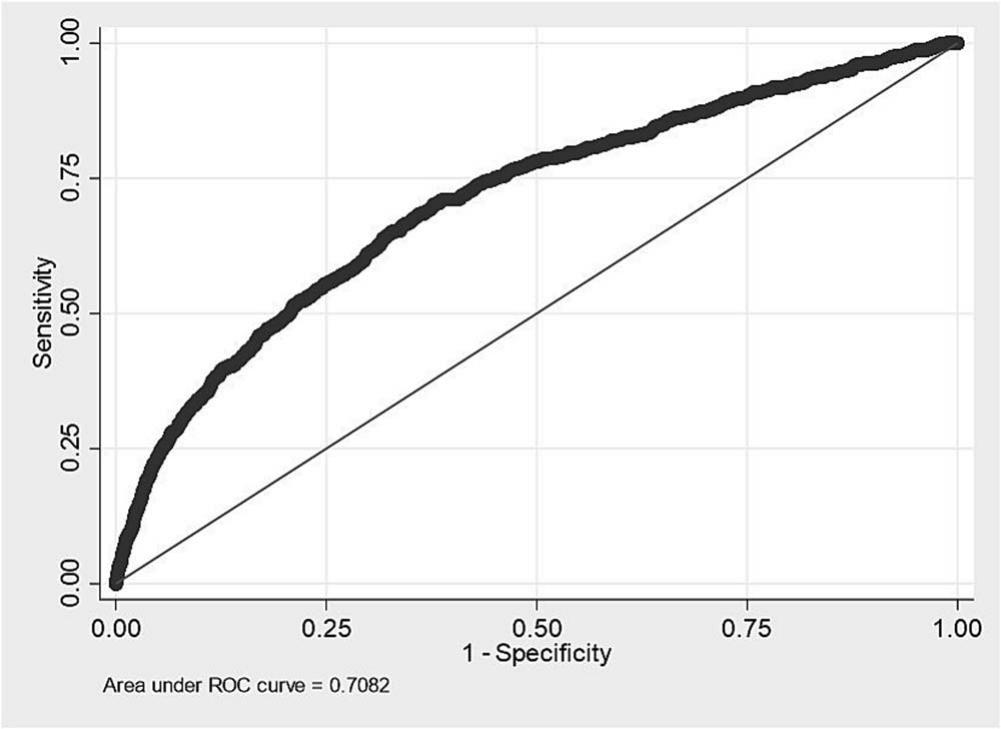
Receiver operating characteristic curve after logistic recalibration (development dataset).

**Figure 2:**
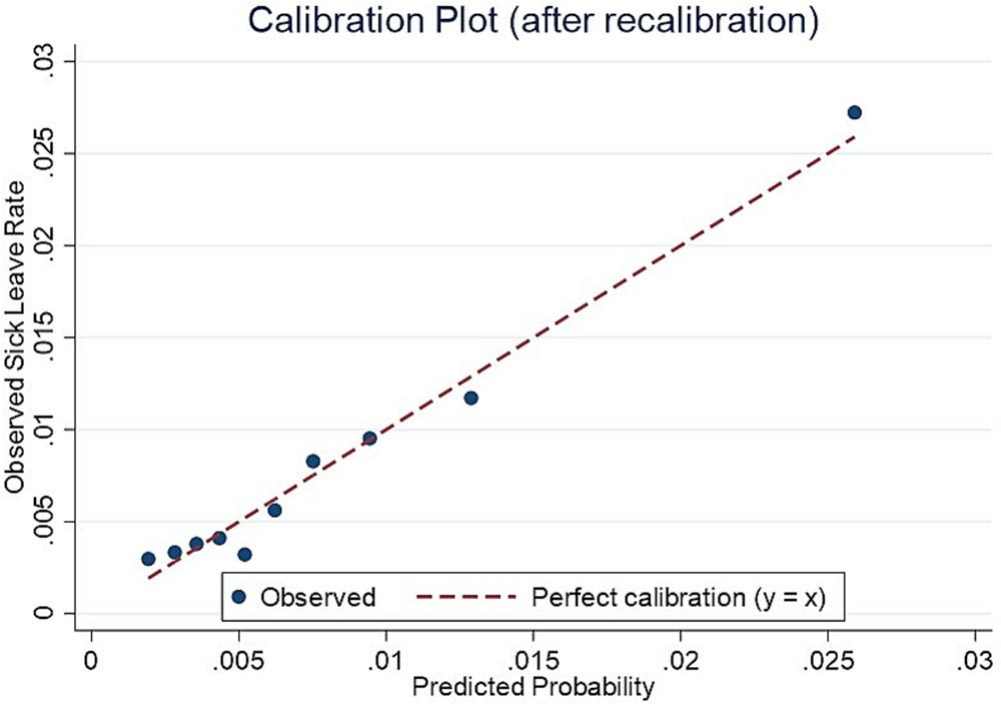
Observed sick-leave rates versus recalibrated predicted probabilities by decile (development dataset).

Figure 1 presents the receiver operating characteristic (ROC) curve of the recalibrated model. The x-axis represents 1 − specificity, and the y-axis represents sensitivity. The AUC was 0.708 (95% CI, 0.688-0.729), and the Brier score was 0.0079, indicating good discrimination and low prediction error. Figure 2 shows the calibration plot after recalibration, demonstrating good agreement between predicted and observed probabilities across all deciles, with points closely aligned along the 45° line. The optimal cutoff point determined by the Youden index was 0.008, with a sensitivity of 66.6%, specificity of 65.3%, and a Youden index of 0.32. In addition, the optimal cutoff point based on the F1 score was 0.023, corresponding to a precision of 2.9%, recall of 26.5%, and an F1 score of 0.052.

### 3.4. Temporal validation (2024)

In the 2024 validation dataset, the model demonstrated high discrimination and good overall accuracy, with an AUC of 0.819 (95% CI, 0.762-0.875) and a Brier score of 0.0026 (Table 3). This performance was substantially better than that of the conventional high-stress classification (AUC, 0.628; 95% CI, 0.611-0.645; Brier score, 0.007).

**Table 3:** Overall predictive performance in the validation dataset (2024).

Model	Dataset	AUC^a^ (95% CI)	Brier score^b^
**Prediction model**	Validation 2024	0.819 (0.762-0.875)	0.0026
**High-stress classification**	Validation 2024	0.628 (0.611-0.645)	0.007

Abbreviation: AUC, area under the receiver operating characteristic curve.

High stress was defined according to the national criterion of the Japanese Stress Check program.

^a^AUC reflects discrimination ability, with higher values indicating better separation between employees with and without subsequent sickness absence.

^b^The Brier score measures overall prediction error, with lower values indicating better calibration and accuracy.

**Table 4:** Classification performance at selected cutoffs in the validation dataset (2024).

Method	Cutoff criterion	Cutoff value	Sensitivity	Specificity	**Youden index** ^**a**^	**Precision** ^**b**^	**F1 score** ^**c**^
**Prediction model**	Youden optimized	0.008	0.825	0.708	0.533	0.007	0.014
**Prediction model**	F1 optimized	0.023	0.193	0.962	0.155	0.013	0.024
**High-stress classification**	National criterion	—	0.509	0.893	0.401	0.012	0.024

Abbreviation: BJSQ, Brief Job Stress Questionnaire.

High stress was defined according to the official criteria of the Japanese Ministry of Health, Labour and Welfare Stress Check program using the 23-item short-form BJSQ (summed-score method).

^a^The Youden index is defined as sensitivity + specificity −1 and reflects the balance between sensitivity and specificity (range: −1 to 1, higher values indicate better overall classification performance).

^b^Precision (positive predictive value) is the proportion of predicted high-risk employees who actually experienced sickness absence.

^c^The F1 score is the harmonic mean of precision and recall and reflects performance for imbalanced outcomes, with higher values indicating better balance between case detection and false positives.

To enable a practical comparison with the conventional classification, cutoff-dependent performance was additionally evaluated (Table 4). At the Youden-optimized cutoff (0.008), the model achieved a sensitivity of 0.825 and a specificity of 0.708 (Youden index, 0.533). At the F1-optimized cutoff (0.023), sensitivity was 0.193 and specificity was 0.962 (F1 score, 0.024). By comparison, the conventional high-stress classification showed a sensitivity of 0.509 and a specificity of 0.893 (Youden index, 0.401; F1 score, 0.024).

## 4. Discussion

This study developed a model to predict mental health–related sick-leave risk using Stress Check and personnel data from a large company with diverse occupational groups. Seven significant predictors were identified: younger age, anxiety, depression, sleep disturbance, low job control, poor job suitability, and low coworker support. The model showed good discrimination (AUC = 0.708) and calibration accuracy (Brier score = 0.0079) in the development phase. In the validation phase, predictive performance further improved (AUC = 0.819; Brier score = 0.0026), indicating stable predictive performance over time and across datasets. By contrast, the conventional high-stress classification demonstrated poor performance (AUC = 0.628; Brier score = 0.007), suggesting that a simple dichotomous screening based on high-stress criteria is insufficient for accurately identifying employees at elevated risk of mental health–related sick leave. These findings highlight the value of implementing more sophisticated, data-driven risk models within Japan’s Stress Check system to support early detection and preventive occupational health interventions.

Among the 7 predictors, anxiety, depression, and sleep disturbance correspond to the “stress response” domain of the BJSQ and have been consistently linked with future sick leave and mental health problems in prior studies.^18,19^ In addition, younger age, low job control, poor coworker support, and poor job suitability have been reported as psychosocial risk factors associated with mental health deterioration and absenteeism.^20–24^ Younger workers may have limited coping experience or resilience under stress, whereas low job control can lead to feelings of helplessness and increased strain. Similarly, lack of coworker support may intensify social isolation, and poor job–person fit can diminish motivation and work engagement—together contributing to elevated psychological distress.

Kawamura et al^15^ previously developed a predictive model for long-term mental health–related sick leave among university staff using BJSQ data, achieving an AUC of 0.768. However, their study was limited to academic staff in a single occupational sector, leaving uncertainty regarding generalizability to broader working populations. The present study extends this work by developing and validating a model in a large, multisector industrial group encompassing diverse job types and work environments. Notably, our model achieved comparable or higher discrimination (AUC = 0.819) even in external validation, supporting its robustness and applicability across different occupational contexts. This suggests that the Stress Check–based prediction model could serve as a practical and scalable approach for mental health risk management in a wide range of industries.

Importantly, the primary contribution of this study lies not in the identification of novel risk factors or the development of new algorithms, but in demonstrating a reproducible and transferable process for developing and validating a sickness-absence risk prediction model using Stress Check data that are routinely collected within an existing legal and organizational framework.

Specifically, this study extends prior work by: (1) adopting a longitudinal cohort design based on annual Stress Check assessments; (2) defining a consistent and operationally meaningful prediction target—namely, sickness absence occurring within 1 year after each Stress Check assessment; (3) validating the model in a large, multisector corporate group encompassing diverse occupations and work environments; and (4) explicitly comparing model-based risk stratification with the conventional high-stress classification used in national policy.

### 4.1. Practical implications

The predictive model developed in this study provides a practical and operationalizable way to translate routinely collected Stress Check data into actionable risk stratification within existing occupational health systems. This enables occupational health professionals to prioritize employees according to predicted risk and to allocate limited resources more efficiently.

For example, workplaces may designate employees with a predicted probability ≥2.3% (the F1-optimized cutoff) for priority follow-up, or—depending on staffing—focus on the top 30 highest-risk employees for targeted interviews and monitoring. Providing individualized, visually presented risk information may also enhance employees’ awareness of their own mental health risks and motivate help-seeking, which is particularly valuable for high-stress employees who do not wish to undergo a physician interview.^25,26^ Furthermore, this risk-stratified feedback can be integrated into other routine occupational health activities—such as post-exam follow-up after mandatory periodic health examinations or assessments of employees working long hours—thereby expanding opportunities for earlier intervention.

Additionally, the 7 predictors identified in this model may serve as a useful educational resource for supervisors. By understanding these risk indicators, supervisors can better recognize early signs of mental distress among subordinates, collaborate with occupational health professionals, and help prevent sick leave through timely support.

### 4.2. Limitations

This study has several limitations. First, this study used the 23-item BJSQ short-form criteria rather than the full 57-item version. This approach aligns with national guidance and common workplace practice, minimizes respondent burden, supports high participation in annual screening, and retains the core psychosocial constructs relevant to mental health. Nevertheless, some extended domains—such as anger–irritability and family support—were not captured, and incorporating these factors in future models may further improve prediction accuracy. Second, although this study focused on Stress Check variables, incorporating additional information such as prior sickness-absence history could further enhance predictive performance. In this study, we could not fully identify or exclude employees with prior mental health–related sickness absence. Although excluding such individuals would be preferable in a strictly incident cohort design, this information is not always systematically available in real-world occupational settings. Our analysis therefore reflects a pragmatic implementation context and should be interpreted as predicting future sickness-absence risk rather than strictly incident cases. Third, although the model demonstrated acceptable discrimination and calibration, recall (26.5%) and precision (2.9%) remained limited. However, such trade-offs are common in predictive modeling of rare outcomes, such as long-term mental health–related sickness absence, and do not necessarily diminish the model’s practical value.^27^ Finally, this study was conducted within a single corporate group in Japan, primarily comprising companies with a predominantly male workforce. Therefore, the generalizability of the findings to other industries, organizational structures, workforce compositions, or cultural contexts should be interpreted with caution and examined in future research.

## 5. Conclusions

This study developed and validated a model to predict mental health–related sick leave using Stress Check and personnel data. The model demonstrated high discrimination and calibration, outperforming the conventional high-stress classification in practical utility. The identified predictors were consistent with previous evidence, supporting their theoretical validity. Within the existing framework of Japan’s Stress Check program, this model may serve as a practical tool for early identification of employees at elevated risk of sick leave and for prioritizing occupational health support and preventive interventions.

## Supplementary Material

Supplementary_materials_uiag011

## Data Availability

The data underlying this article are available from the corresponding author upon reasonable request.
